# Genome-wide analysis yields new loci associating with aortic valve stenosis

**DOI:** 10.1038/s41467-018-03252-6

**Published:** 2018-03-07

**Authors:** Anna Helgadottir, Gudmar Thorleifsson, Solveig Gretarsdottir, Olafur A. Stefansson, Vinicius Tragante, Rosa B. Thorolfsdottir, Ingileif Jonsdottir, Thorsteinn Bjornsson, Valgerdur Steinthorsdottir, Niek Verweij, Jonas B. Nielsen, Wei Zhou, Lasse Folkersen, Andreas Martinsson, Mahyar Heydarpour, Siddharth Prakash, Gylfi Oskarsson, Tomas Gudbjartsson, Arnar Geirsson, Isleifur Olafsson, Emil L. Sigurdsson, Peter Almgren, Olle Melander, Anders Franco-Cereceda, Anders Hamsten, Lars Fritsche, Maoxuan Lin, Bo Yang, Whitney Hornsby, Dongchuan Guo, Chad M. Brummett, Gonçalo Abecasis, Michael Mathis, Dianna Milewicz, Simon C. Body, Per Eriksson, Cristen J. Willer, Kristian Hveem, Christopher Newton-Cheh, J. Gustav Smith, Ragnar Danielsen, Gudmundur Thorgeirsson, Unnur Thorsteinsdottir, Daniel F. Gudbjartsson, Hilma Holm, Kari Stefansson

**Affiliations:** 1 deCODE genetics/Amgen Inc., Reykjavik, 101 Iceland; 2 Faculty of Medicine, University of Iceland, Reykjavik, 101 Iceland; 3 Department of Cardiology, University of Groningen, University Medical Center Groningen, 9700 RB Groningen, The Netherlands; 4 Medical and Population Genetics Program, Broad Institute of MIT and Harvard, Cambridge, 02142 MA USA; 5 Department of Internal Medicine, Division of Cardiovascular Medicine, University of Michigan, Ann Arbor, 48109 MI USA; 6 Department of Computational Medicine and Bioinformatics, University of Michigan, Ann Arbor, 48109 MI USA; 7 Cardiovascular Medicine Unit, Department of Medicine, Karolinska University Hospital Solna, Karolinska Institutet, Stockholm, 17176 Sweden; 8 Department of Bioinformatics, Technical University of Denmark, Copenhagen, 2800 Denmark; 9 Department of Cardiology, Clinical Sciences, Lund University and Skåne University Hospital, Lund, 22185 Sweden; 10 Department of Anesthesiology, Perioperative and Pain Medicine, Brigham and Women’s Hospital, 75 Francis Street, Boston, MA 02115 USA; 11 Department of Internal Medicine, Division of Medical Genetics, University of Texas Health Science Center at Houston, Houston, 77030 TX USA; 12 Childrens Hospital, Landspitali National University Hospital of Iceland, Reykjavik, 101 Iceland; 13 Department of Surgery and Cardiothoracic Surgery, Landspitali National University Hospital, Reykjavik, 101 Iceland; 14 Section of Cardiac Surgery, Department of Surgery, Yale University School of Medicine, New Haven, 06510 CT USA; 15 Department of Clinical Biochemistry, Landspitali National University Hospital, Reykjavik, 101 Iceland; 16 Heilsugaeslan Solvangi, Hafnarfjördur, 220 Iceland; 17 Department of Family Medicine, University of Iceland, Reykjavik, 101 Iceland; 18 Department of Clinical Sciences, Lund University, Malmö, 22185 Sweden; 19 Department of Internal Medicine, Skåne University Hospital, Malmö, 22185 Sweden; 20 Cardiothoracic Surgery Unit, Department of Molecular Medicine and Surgery, Karolinska University Hospital Solna, Karolinska Institutet, Stockholm, 17176 Sweden; 21 HUNT Research Centre, Department of Public Health and General Practice, Norwegian University of Science and Technology, Levanger, 7491 Norway; 22 K.G. Jebsen Center for Genetic Epidemiology, Department of Public Health, Norwegian University of Science and Technology, Trondheim, 7491 Norway; 23 Department of Cardiac Surgery, University of Michigan, Ann Arbor, MI 48105 USA; 24 Frankel Cardiovascular Center, University of Michigan, Ann Arbor, MI 48109 USA; 25 Department of Anesthesiology, University of Michigan, Ann Arbor, MI 48105 USA; 26 Department of Biostatistics, University of Michigan, Ann Arbor, MI 48109 USA; 27 Medicine Services, Texas Heart Institute, St. Luke’s Episcopal Hospital, Houston, TX 77030 USA; 28 Department of Human Genetics, University of Michigan, Ann Arbor, MI 48109 USA; 29 Massachusetts General Hospital, Harvard Medical School, Broad Institute of Harvard and MIT, Boston, MA 02114 USA; 30 Cardiovascular Research Center, Massachusetts General Hospital, Boston, MA 02114 USA; 31 Department of Internal Medicine, Division of Cardiology, Landspitali National University Hospital of Iceland, Reykjavik, 101 Iceland; 32 School of Engineering and Natural Sciences, University of Iceland, Reykjavik, 101 Iceland

## Abstract

Aortic valve stenosis (AS) is the most common valvular heart disease, and valve replacement is the only definitive treatment. Here we report a large genome-wide association (GWA) study of 2,457 Icelandic AS cases and 349,342 controls with a follow-up in up to 4,850 cases and 451,731 controls of European ancestry. We identify two new AS loci, on chromosome 1p21 near *PALMD* (rs7543130; odds ratio (OR) = 1.20, *P* = 1.2 × 10^−22^) and on chromosome 2q22 in *TEX41* (rs1830321; OR = 1.15, *P* = 1.8 × 10^−13^). Rs7543130 also associates with bicuspid aortic valve (BAV) (OR = 1.28, *P* = 6.6 × 10^−10^) and aortic root diameter (*P* = 1.30 × 10^−8^), and rs1830321 associates with BAV (OR = 1.12, *P* = 5.3 × 10^−3^) and coronary artery disease (OR = 1.05, *P* = 9.3 × 10^−5^). The results implicate both cardiac developmental abnormalities and atherosclerosis-like processes in the pathogenesis of AS. We show that several pathways are shared by CAD and AS. Causal analysis suggests that the shared risk factors of Lp(a) and non-high-density lipoprotein cholesterol contribute substantially to the frequent co-occurence of these diseases.

## Abstract (web summary)

Aortic valve stenosis (AS) is the most common valvular heart disease. Here the authors identify two new AS loci that also associate with bicuspid aortic valve, aortic root diameter and/or coronary artery disease implicating both developmental abnormalities and atherosclerosis-like processes in AS.

## Introduction

Aortic valve stenosis (AS) is characterized by thickened and calcified valvular cusps causing left ventricular outflow obstruction. This progressive disease is usually graded as mild, moderate, or severe, based on the valve area and pressure gradient across the valve. Severe AS is a notable cause of morbidity and mortality, affecting approximately 5% of those over 70 years of age^1–3^, and the estimated 5-year survival in symptomatic severe AS ranges from 15 to 50% unless outflow obstruction is relieved by aortic valve replacement^3^.

The pathogenesis of the disease remains poorly understood. However, several of the associated clinical risk factors of calcified aortic valve are shared by atherosclerotic disease, and immunohistochemical studies show that calcified aortic valve lesions have many characteristic features of atherosclerosis, including initial endothelial damage, oxidized lipid deposition, chronic inflammation, and calcification^4^. In addition, bicuspid aortic valve (BAV), the most common congenital cardiac malformation, when the aortic valve has two leaflets instead of three, accelerates the development of AS by decades^4^. While the prevalence of BAV is 0.5–2% in the population, BAV is found in up to half of those with severe AS^5^.

Little is known about the genetics of AS, although a recent genome-wide association (GWA) study reported the association of rs10455872 in the *LPA* gene, encoding apolipoprotein(a) of lipoprotein (a) (Lp(a)), with calcification of the aortic valve, and with AS^6^. Elevated serum levels of Lp(a) have also been associated with increased risk of AS^7^. These findings are in keeping with a common pathogenic feature of AS and atherosclerosis^8,9^.

Another genetic study recently showed that a rare p.Arg721Trp *MYH6* missense variant, which was previously shown to associate with sick sinus syndrome and atrial fibrillation^10,11^, also associates with coarctation of the aorta, BAV, and with AS^12^.

Here, we describe a large GWA study of AS including 2,457 cases and 349,342 controls, with follow-up in up to 4,850 AS cases and 451,731 controls. We examined the association of AS variants with BAV and several other cardiovascular conditions and assessed the shared genetic risk of AS and coronary artery disease (CAD).

## Results

### Novel variants associate with aortic stenosis

We tested 32.5 million sequence variants for association with AS in 2,457 Icelandic cases and 349,342 controls (see Manhattan plot in Supplementary Fig. 1). We identified the variants by whole-genome sequencing 15,220 Icelanders, and imputed them into 151,678 chip-typed, long-range phased individuals and their close relatives^13^.

We observed one genome-wide significant association, between AS and the intergenic variant rs7543130 (effect allele frequency (EAF) [A] = 51.2%) on chromosome 1p21 near the *PALMD* gene (odds ratio (OR) = 1.23; 95% confidence interval (CI): 1.15–1.31, *P* = 6.8 × 10^−10^ (significance threshold for intergenic variants set at *P* = 7.9 × 10^−10^, see Methods and ref. ^14^)) (Table 1). We noted that rs7543130 was recently reported to associate with aortic root size^15^ and we replicate this association in our Icelandic aortic root dimension sample (*P* = 1.3 × 10^−8^) (Table 2).

**Table 1:** Meta-analysis results for aortic valve stenosis variants

	Cases/controls	*PALMD* intergenic rs7543130 [A/C] EAF = 51.2%		*TEX41* intronic rs1830321 [T/C] EAF = 37.5%		*LPA* intronic rs10455872 [G/A] EAF = 6.2%	
		OR (95% CI)	*P* value	OR (95% CI)	*P* value	OR (95% CI)	*P* value
Iceland	2457/349,342	1.23 (1.15–1.31)	6.8 × 10^−10^	1.20 (1.12–1.28)	7.6 × 10^−8^	1.4 (1.23–1.56)	1.8 × 10^−7^
Sweden (MDCS)^a^	470/15,162	1.14 (0.98–1.33)	0.092	1.21 (1.05–1.39)	0.0080	1.55 (1.28–1.88)	1.0 × 10^−5^
Sweden, Stockholm	318/1376	1.25 (0.98–1.59)	0.068	1.18 (0.89–1.56)	0.24	1.19 (0.90–1.57)	0.23
UK Biobank	1844/406,814	1.25 (1.17–1.33)	3.8 × 10^−11^	1.14 (1.06–1.22)	1.36 × 10^−4^	1.54 (1.38–1.71)	4.8 × 10^−15^
Norway (HUNT)	1546/24,235	1.13 (1.05–1.22)	0.0012	1.11 (1.02–1.20)	0.010	1.48 (1.28–1.71)	1.0 × 10^−7^
USA, Michigan	251/2510	1.15 (0.96–1.39)	0.13	1.01 (0.85–1.24)	0.76	1.32 (0.94–1.84)	0.10
Combined	6886/799,439	1.20 (1.16–1.25)	1.2 × 10^−22^	1.15 (1.11–1.20)	1.8 × 10^−13^	1.46 (1.37–1.56)	1.9 × 10^−31^

Results are shown for the discovery and follow-up datasets and the joint analysis (combined). The effect allele is the first allele in brackets [effect allele/non-effect allele]. The EAF is for the Icelandic population. *P* value from logistic regression analysis. Results from the different study groups were combined using a Mantel–Haenszel model

*EAF* effect allele frequency, *OR* allelic odds ratio, 95% *Cl* 95% confidence interval, *MDCS* Malmö Diet and Cancer study

^a^ The association results for the rs10455872 variant in the MDCS included 613 cases and 28,109 controls

**Table 2:** Association of aortic valve stenosis variants with other cardiovascular traits

Bicuspid aortic valve	208/25,139	*PALMD* intergenic rs7543130 [A/C]		*TEX41* intronic rs1830321 [T/C]		*LPA* intronic rs10455872 [G/A]		*MYH6* missense rs387906656 [A/G] p.Arg721Trp	
		OR (95% CI)	*P* value	OR (95% CI)	*P* value	OR (95% CI)	*P* value	OR (95% CI)	*P* value
Bicuspid aortic valve	208/25,139	1.26 (0.99, 1.60)	0.059	1.31 (1.03, 1.67)	0.025	1.12 (0.72, 1.75)	0.61	8.04 (3.36, 19.22)	2.8 × 10^−6^
*Sweden-Stockholm*	275/1516	1.27 (1.06, 1.52)	0.0098	1.20 (0.99, 1.46)	0.063	1.02 (0.92, 1.12)	0.77		
*USA-Houston*	147/864	1.29 (0.99, 1.67)	0.057	1.25 (0.97, 1.60)	0.085	0.96 (0.51, 1.81)	0.91		
*USA-Boston*	452/1834	1.27 (1.09, 1.48)	0.002	1.10 (0.95, 1.28)	0.21	1.44 (1.08, 1.93)	0.014		
*USA-Michigan*	473/4730	1.31 (1.14, 1.51)	1.2 × 10^−4^	1.00 (0.86, 1.16)	0.97	1.19 (0.93, 1.54)	0.17		
*Combined BAV*	1555/33,883	1.28 (1.19, 1.39)	6.6 × 10^−10^	1.12 (1.04, 1.22)	5.3 × 10^−3^	1.07 (0.98, 1.16)	0.13		
Atrial septal defect	708/353,019	1.23 (1.07, 1.42)	3.9 × 10^−3^	1.22 (1.06, 1.41)	5.9 × 10^−3^	1.15 (0.87, 1.52)	0.32	3.17 (1.47, 6.81)	3.2 × 10^−3^
Ventricular septal defect	902/357,428	1.23 (1.07, 1.42)	4.8 × 10^−3^	1.04 (0.90, 1.21)	0.59	1.14 (0.84, 1.53)	0.41	4.40 (2.14, 9.07)	5.7 × 10^−5^
Coronary artery disease	37,782/318,845	1.00 (0.97, 1.02)	0.74	1.05 (1.03, 1.08)	9.3 × 10^−5^	1.28 (1.21, 1.34)	2.4 × 10^−22^	1.21 (1.00, 1.48)	0.056
Phenotype (qtl)	*N*	β (SE)	*P* value	β (SE)	*P* value	β (SE)	*P* value	β (SE)	*P* value
Aortic root diameter	19,513	0.065 (0.01)	1.3 × 10^−8^	−0.017 (0.02)	0.16	−0.052 (0.02)	0.020	−0.068 (0.08)	0.40

Association of aortic valve stenosis variants with cardiovascular phenotypes is shown for Icelandic samples. Follow-up and joint analysis (combined BAV) is also provided for the association with BAV. The effect allele is the first allele in brackets [effect allele/non-effect allele]. The effect (*β*) for aortic root diameter is given in standardized units. Logistic (cc) or linear (qtl) regression analyses were used for association testing. Results from the different study groups were combined using a Mantel–Haenszel model

*Cc* case–control, *Qtl* quantitative trait, *OR* allelic odds ratio, *95% Cl* 95% confidence interval, *BAV* bicuspid aortic valve, *SE* standard error

We tested the top seven common and low-frequency variants in the discovery GWA scan, including rs7543130, in up to 4,850 AS cases and 451,731 controls from Sweden, Norway, United Kingdom, and the United States (Table 1, Supplementary Data 1). The joint analysis showed a robust association between AS and rs7543130 (OR = 1.20; 95% CI: 1.16–1.25; *P* = 1.2 × 10^−22^) as well as rs1830321 (EAF[T] = 37.5%) intronic to *TEX41*, a non-protein coding gene on chromosome 2q22 (OR = 1.15; 95% CI: 1.11–1.20, *P* = 1.8 × 10^−13^) (Table 1).

We replicated the reported association of the intronic *LPA* variant^6^ rs10455872 with AS in Iceland and the follow-up sample sets (combined OR = 1.46; 95% CI: 1.37–1.56, *P* = 1.9 × 10^−31^) (Table 1). In contrast, we did not find association with variants implicating osteogenic and calcium signaling pathway genes, previously reported to suggestively associate with AS^16^ (*P* > 0.05 in Iceland and UK Biobank).

We tested the association of the two novel AS variants and the *LPA* variant with a subset of Icelandic AS cases who had undergone aortic valve replacement, representing those with severe AS. Although less significant, likely due to smaller sample size, the effect sizes were not significantly different from those for all AS (Supplementary Data 2).

### Aortic stenosis variants and cardiovascular phenotypes

We tested the rs7543130 near *PALMD*, rs1830321 in *TEX41*, and the *LPA* rs10455872, for association with BAV, a major risk factor for AS^4,5^, in 1,555 cases and 33,883 controls from Iceland, Sweden, and the United States. Both of the novel AS variants associate with BAV and the rs7543130 association was genome-wide significant (OR = 1.28; 95% CI: 1.19–1.39; *P* = 6.6 × 10^−10^; OR = 1.12, 95% CI: 1.04–1.22, *P* = 5.3 × 10^−3^ for rs1830321). The *LPA* rs10455872 does not associate with BAV (Table 2).

Table 2 also shows the association of the rare p.Arg721Trp *MYH6* missense variant rs387906656 (EAF = 0.34%) with the risk of BAV (OR = 8.04; 95% CI: 3.36–19.22; *P* = 2.8 × 10^−6^). This variant was previously shown to associate with sick sinus syndrome and atrial fibrillation^10,11^, and was recently reported also to associate with coarctation of the aorta, BAV, and AS (OR = 2.65; 95% CI: 1.78–3.96; *P* = 1.8 × 10^−6^)^12^. The effect size on BAV is substantially greater than that for AS (*P* = 0.023), suggesting that the AS risk conferred by this variant is mediated through BAV.

Next, we examined the association of the AS variants with several other cardiovascular diseases in Icelandic data. In line with the BAV association of p.Arg721Trp in *MYH6*, rs7543130, and rs1830321, all three variants associate with ventricular defects and/or atrial septal defects (*P* < 0.006) (Table 2 and Supplementary Data 2).

Like the *LPA* variant, rs1830321 in *TEX41* associates with CAD in Iceland (OR = 1.05, 95% CI:1.03–1.08; *P* = 9.3 × 10^−5^), but the *MYH6* missense variant and rs7543130 near *PALMD* do not (Table 2 and Supplementary Data 2). The *TEX41* rs1830321 is in linkage disequilibrium (LD) with a known GWA CAD variant rs2252641 at the same locus (*R*^2^ = 0.80)^17^.

Given that several atherosclerosis risk factors have been associated with AS^6,18,19^, we tested the novel AS variants for association with the traditional cardiovascular risk factors and observed a nominally significant association (*P* < 0.02) between rs1830321 and systolic and diastolic blood pressure in Iceland (Supplementary Data 3) and in data from the UK Biobank (https://biobankengine.stanford.edu/search#).

### Shared genetic risk factors with CAD

The frequent comorbidity of CAD and AS^20^, together with the similarities in histopathology^4^, suggest shared genetic predisposition. Therefore, we tested 71 CAD variants^21,22^ for association with AS, both individually (Supplementary Data 4 and Table 3) and as a weighted genetic risk score (CAD-GRS*-all*) (Table 4). We excluded from this analysis the *LPA* variant rs10455872 and rs1830321 in *TEX41* that associate genome-wide significantly with both CAD and AS.

**Table 3:** Coronary artery disease variants and aortic root size variants that associate with aortic valve stenosis

Primary association	Locus	Chr.	Coding effect	Rs name	EA/other allele	OR (95% CI)	*P* value	*P*_het_	*I* ^2^
CAD	*CELSR2/PSRC1*	1	Downstream gene	Rs646776	T/C	1.11 (1.05–1.18)	3.4 × 10^−4^	0.82	0
CAD	*LPA*	6	Missense (p.Ile1891Met)	Rs3798220	C/T	1.55 (1.33–1.81)	2.1 × 10^−8^	0.4	0
CAD	*SH2B3*	12	Missense (p.Trp60Arg)	Rs3184504	C/T	0.91 (0.87–0.96)	1.6 × 10^−4^	0.94	0
Aortic root size	*CFDP1* ^a^	16	Intronic	Rs17696696	G/T	1.07 (1.03–1.11)	1.3 × 10^−4^	0.055	60.5
CAD	*ANGPTL4* ^b^	19	Missense (p.Glu40Lys)	Rs116843064	A/G	0.77 (0.68–0.88)	9.5 × 10^−5^	0.36	8.6

Shown are CAD variants and aortic root size variants that associate with AS. A total of 71 CAD and 11 aortic root size variants from genome-wide association studies were tested (primary association). The *CELSR2/PSRC1, LPA*, and *SH2B3* variants were tested in 4,301 AS cases and 756,156 controls from Iceland and the UK Biobank. Results from the different study groups were combined using a Mantel–Haenszel model

*P* values for the combined analyses are provided

*CAD* coronary artery disease, *AS* aortic valve stenosis, *Chr.*: chromosome, *EA* effect allele, *OR* odds ratio, *P*_het_: *P* value for heterogeneity between study groups, *I*^*2*^: heterogeneity *I*^2^ statistics for the combined analysis

^a^ The *CFDP1* variant was tested in 6,416 cases and 784,277 controls (additional samples from Sweden-Stockholm, Norway-HUNT, and the USA, Michigan)

^b^ The *ANGPTL4* variant was tested in 6,886 cases and 799,439 controls (same samples as for *CFDP1* plus samples from Sweden-MDCS)

**Table 4:** The association of coronary artery disease genetic risk score with aortic valve stenosis

		Iceland		UK Biobank		Iceland + UK Biobank combined	
		*β*	*P* value	*β*	*P* value	*β* (95% CI)	*P* value
CAD-GRS-*all*	CAD	0.77	5.2 × 10^−153^	0.76	<10^−300^	0.76 (0.73–0.79)	<10^−300^
.	AS	0.29	0.00027	0.28	7.2 × 10^−6^	0.28 (0.19–0.38)	7.5 × 10^−9^
.	AS_adj.CAD_	0.03	0.62	−0.05	0.45	−0.01 (−0.10, 0.08)	0.83
CAD-GRS*-lip*	CAD	0.89	1.4 × 10^−36^	0.92	7.8 × 10^−99^	0.91 (0.84–0.99)	1.4 × 10^−134^
.	AS	1.05	2.3 × 10^−9^	0.99	3.8 × 10^−11^	1.02 (0.79–1.24)	5.1 × 10^−19^
.	AS_adj.CAD_	0.77	1.5 × 10^−5^	0.60	6.6 × 10^−5^	0.67 (0.45–0.90)	5.1 × 10^−9^
CAD-GRS-*non-lip*	CAD	0.73	6.3 × 10^−120^	0.72	4.3 × 10^−293^	0.73 (0.69–0.78)	<10^−300^
.	AS	0.14	0.074	0.14	0.048	0.14 (0.04–0.24)	0.0076
.	AS_adj.CAD_	−0.11	0.15	−0.18	0.0088	−0.15 (−0.25, −0.05)	0.0036

CAD-GRS-*all* (based on 71 reported CAD variants). CAD-GRS-*lip* (based on 14 CAD variants with reported association with LDL cholesterol (or non HDLcholesterol), or variants at the *LPA* locus. GRS-*non-lip* (based on 57 CAD variants, the same as in CAD-GRS*-all*, but excluding variants in CAD-GRS-*lip*). The effects of the GRSs on CAD are shown for comparison. Logistic regression was used for association testing. Results from the different study groups were combined using a Mantel–Haenszel model

Number of cases–controls in Iceland and UK Biobank, respectively: CAD = 17,488/124,620 and 26,384/382,294; AS = 1,591/140,517 and 1,844/406,814

*P* value represented as <10^−300^ is <1 × 10^−300^

*GRS* genetic risk score, *CAD* coronary artery disease, *AS* aortic valve stenosis

In the Icelandic and UK Biobank datasets combined, four CAD variants associate with AS at a significance threshold set at *P* = 7.0 × 10^−4^ = 0.05/71. These are the *LPA* variant rs3798220 (p.Ile1891Met), rs116843064 in *ANGPTL4 *(p.Glu40Lys), rs646776 at the *CELSR2*/*PSRC1* locus, and rs3184504 in *SH2B3* (p. Trp60Arg) (Table 3 and Supplementary Data 4 and 5).

Consistent with a shared genetic risk, the CAD-GRS-*all* associates with AS both in the Icelandic and the UK Biobank datasets (combined *P* = 7.5 × 10^−9^) (Table 4). However, the effect on AS is only 37% of the effect on CAD and the AS association is  not significant after adjustment for CAD diagnosis. Given the reported association between genetic predisposition to both elevated Lp(a) and low-density lipoprotein (LDL) cholesterol and AS^6,19^, we tested a subset of CAD-GRS (labeled as CAD-GRS*-lip*), constructed based on 14 Lp(a) and LDL cholesterol/non-high-density lipoprotein (HDL) cholesterol variants (Supplementary Data 4) for association with AS. The CAD-GRS*-lip* associated strongly with AS (*P* = 5.1 × 10^−19^) with an effect that was similar or larger than that for CAD (*β* = 0.91 and 1.02, for CAD and AS, respectively). This association with AS remained after adjusting for CAD diagnosis (*P* = 5.1 × 10^−9^) (Table 4), suggesting that the genetic predisposition to elevated Lp(a) and LDL cholesterol explains in large part the shared genetic risk between CAD and AS. Specifically, examining the impact of CAD-GRS-*lip* on the risk of AS among CAD cases shows that CAD cases with genetic predisposition to high Lp(a) or LDL/non-HDL cholesterol are at a greater risk of having AS than CAD cases without such predisposition (*P* = 8.5 × 10^−7^) (Supplementary Data 6). In contrast, the complementary subset of CAD-GRS-all (CAD-GRS-*non-lip*), in which the Lp(a) and LDL/non-HDL cholesterol variants are excluded, associates with less risk of AS after adjusting for CAD status (*P* = 0.0036) (Table 4).

### Aortic root size variants and aortic stenosis

Given the known association of rs7543130[A] on chromosome 1p21 with increased aortic root dimension^15^ and the recognized relationship between BAV and aortopathy^5,23^, we excluded known or suspected BAV cases from our echocardiogram database and re-examined the association of this variant with aortic root size. The association with aortic root size remained in this data (*β* = 0.062, *P* = 4.4 × 10^−8^) (Supplementary Data 2).

We then tested 11 other reported aortic root size variants^15^ for association with AS in Icelandic and UK Biobank datasets (Supplementary Data 7). One of these variants, rs17696696[G] intronic to *CFDP1*, associates with AS in these samples and was thus tested in additional 2,115 AS cases and 28,121 controls; the joint analysis yielded OR = 1.07, 95% CI: 1.03–1.11, *P* = 0.00013 (Table 3). A correlated variant rs4888378 (*R*^2^ = 0.98) has been reported to associate with carotid intima–media thickness and with the risk of CAD^24^. We also observed a previously unreported genome-wide significant association with CAD in Iceland and the UK Biobank data (combined OR for rs17696696[G] = 1.05, 95% CI: 1.03–1.07, *P* = 1.4 × 10^–10^). The AS and CAD risk allele of rs17696696[G] in *CFDP1* associates with smaller aortic root diameter. None of the other AS variants associated with aortic root size (Table 2).

### Candidate causal variants and genes

Attempting to identify candidate causal variants and genes at the *PALMD* and *TEX41* loci, we first looked for association of the AS variants with expression quantitative trait loci (eQTL) using the Genotype-Tissue Expression dataset^25^. Assessment of 44 diverse human tissues from adults indicated association of rs1830321 with *TEX41* expression, albeit limited to thyroid tissue, but no eQTLs were observed for rs7543130.

To further investigate potential functional relevance of the two AS variants, we mapped variants in LD (*R*^2^ > 0.5) with rs7543130 near *PALMD* and rs1830321 in *TEX41* to regulatory regions in heart and aorta tissue samples using public data from the NIH Roadmap Epigenomics Consortium^26,27^. Subsequently, we used chromatin interaction maps^28^ for aorta and left and right ventricular heart tissue samples to look for interactions between the regulatory regions, to which AS risk variants mapped, and gene promoters.

**Fig. 1:**
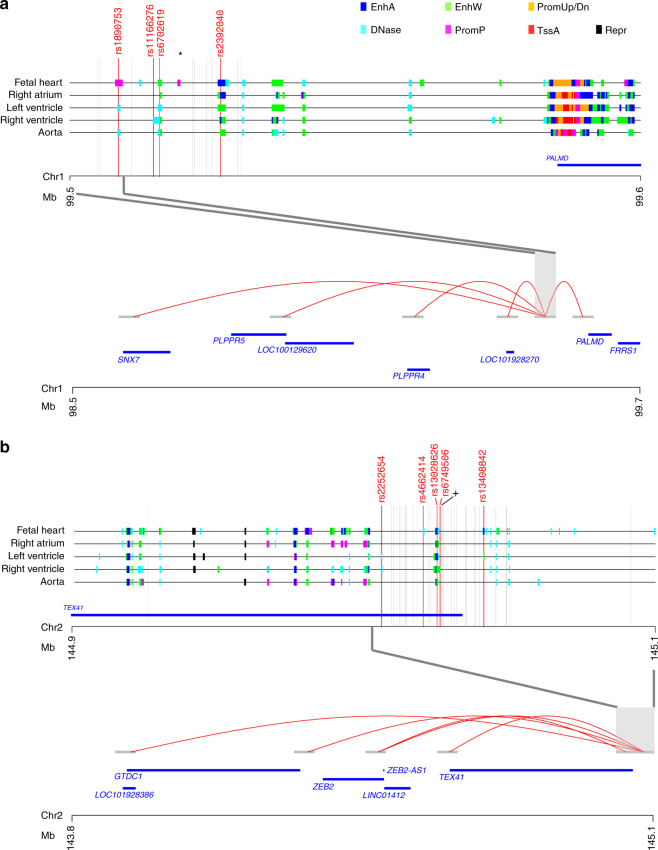
Chromatin interactions between regulatory regions harboring candidate causal variants at the *PALMD* and *TEX41* loci. Chromatin states indicative of regulatory regions for the aortic valve stenosis locus on chromosomes 1p21 (**a**) and 2q22 (**b**) are shown for heart and aorta tissue samples. Different types of regulatory states are indicated with distinct colors shown at the top of the figure. EnhA (Enhancer Active), EnhW (Enhancer Weak), PromUp/Dn (Chromatin marks characteristic of a promoter region found upstream or downstream of TSS), DNase (DNase, nucleosome-free/open chromatin region), PromP (Promoter poised region, marked simultaneously as active and repressed, poised for activation during development), TssA (Transcription Start Site, Activated), and Repr (Repressive marks, heterochromatin). Vertical gray lines indicate the variants found in LD (*R*^2^ > 0.50) with **a** rs7543130 (*) (*N* = 19) or **b** rs1830321 (+) (*N* = 50). Variants found to overlap with regulatory regions in any of the five tissues are marked up and indicated as red vertical lines. Long-range chromatin interactions in left ventricle tissue samples are shown for **a** the region harboring rs1890753 on chromosome 1p21 with red curved lines, including interactions to promoters for *PALMD*, *PLPPR4*, *PLPPR5*, *DPH5* and *SNX7*, *LOC100129620* and *LOC101928270*, and for **b** regions harboring rs13028626, rs6749506, rs2252654, rs4662414, and rs13408842 that directly interact with the promoter regions of *ZEB2*, *GTDC1*, *ZEB2-AS1*, *LINC01412* and *TEX41*

At the *PALMD* locus, four variants (rs11166276, rs6702619, rs1890753, and rs2392040) mapped to three distinct regulatory regions annotated as enhancers and poised promoter (Fig. 1a, upper panel). Multiple chromatin interactions were observed for the regulatory regions harboring these four variants in left ventricular samples. Notably, only the region harboring rs1890753 (*R*^2^ = 0.97 with rs7543130) interacted with promoters of genes (Fig. 1a, lower panel). Thus, rs1890753 represents the candidate causal variant at the chromosome 1p21 locus, by directly interacting with the promoters of *PALMD*, *SNX7*, *PLPPR5*, and *PLPPR4*, and the non-coding RNAs, *LOC100129620* and *LOC101928270*. In fetal heart tissue, a poised promoter state is found at the rs1890753 locus (Fig. 1a, upper panel).

At the *TEX41* locus, five variants in LD with rs1830321 overlapped with four distinct regulatory regions (Fig. 1b, upper panel). Chromatin interaction mapping in left ventricular tissue identified the regulatory regions harboring all five variants (rs13028626, rs6749506, rs2252654, rs4662414, and rs13408842) in direct contact to the promoter region of *ZEB2*, and the non-coding RNAs *ZEB2-AS1* and *LINC01412* (Fig. 1b, lower panel). In addition, the rs13408842 region directly interacted with the promoter of *GTDC1* and the non-coding RNA genes *TEX41* and *LOC101928386*. Pairwise correlations (*R*^2^) between the five variants and the lead variant rs1830321 ranged from 1.0 for rs13028626, to 0.61 for rs2252654.

Chromatin interactions between the regulatory regions harboring candidate causal variants at the *PALMD* and *TEX41* loci were much less frequent in right ventricular tissue and aorta, compared with the left ventricle, and none overlapped with gene promoters (Supplementary Fig. 2).

## Discussion

Through a large GWA study, we have discovered two common AS variants on chromosomes 1p21 near *PALMD* and 2q22 in *TEX41*, and replicated the previously reported AS variant in *LPA*^6^. Like the rare AS variant in *MYH6*^12^, both of the novel AS variants also associate with BAV and congenital cardiac septal defects. The chromosome 2q22 variant also associates with CAD risk.

Given that BAV is a major risk factor for AS, and that the *MYH6* and chromosome 1p21 variants have substantially greater effects on BAV than on AS, it may be postulated that the AS risk conferred by these variants is mediated through BAV. However, as we have limited information on whether AS occured on the background of bicuspid or tricuspid valve, we were not able to determine whether these variants associate with AS in the absence of BAV.

Interestingly, the AS and BAV risk allele of rs7543130 near *PALMD* also associates with increased aortic root size. The relationship between BAV and aortopathy is well recognized and several studies suggest that the dilation of the proximal ascending aorta results from changes in flow secondary to the presence of BAV^5,23,29^. This raises the question whether the effect of chromosome 1p21 variant on aortic root size can be explained by its association with BAV. However, our results indicate that the variant’s impact on aortic root size is not merely a consequence of BAV, since excluding BAV cases from the analysis had minimal effect on the association. We also note that the *MYH6* missense variant has a large effect on BAV but no effect on aortic root size. We did not find a consistent relationship between genetic associations with risk of AS and aortic root size, but found that one additional aortic root size variant, rs17696696 intronic to *CFDP1*, associates with AS.

We demonstrate that this new AS variant, rs17696696 in *CFDP1*, associates genome-wide significantly with CAD, like rs1830321 near *TEX41* and the *LPA* rs10455872 (ref. ^6^). Further, we found that four other CAD variants associate with AS, supporting the notion that there may be a cause shared by CAD and AS. However, contesting a generalized common pathophysiology, causal analysis suggests that only some genetic pathways are shared by CAD and AS, and that the risk of both diseases conferred by Lp(a) and LDL/non-HDL cholesterol levels contributes substantially to the frequent co-occurence of these two diseases.

These results support the assumption that lowering Lp(a) and non-HDL cholesterol levels might slow or prevent progression of AS. However, in a randomized trial of 1,873 patients with mild-to-moderate AS who received statin plus ezetimibe therapy or placebo, active therapy did not reduce the composite outcome of combined aortic valve events and ischemic events during a median follow-up of 52.2 months^30^. It remains conceivable that non-HDL cholesterol lowering therapy implemented earlier in the disease process could affect development of AS. Other potential therapeutic interventions such as Lp(a) lowering among those with high Lp(a) levels need to be explored.

Although we cannot establish how the AS variants at *PALMD* and *TEX41* affect the pathogenesis of disease, chromatin conformational experiments provide clues about potential mechanisms. These experiments show folding of chromatin such that distinct regulatory regions harboring variants in high LD with the lead AS variants, physically interact with several gene promoters, suggesting several candidate causal genes at both loci. Interestingly, in line with an impact during fetal development, a poised promoter state was found in fetal heart tissue for a candidate causal variant rs1890753 at chromosome 1p21. Poised promoters are considered to be involved in the expression of developmental genes allowing for a rapid response to differentiation signals.

At the *TEX41* locus, we note that *ZEB2*, one of the genes suggested through chromatin interaction studies, is a strong biological candidate. ZEB2 is a DNA-binding transcriptional repressor that interacts with activated SMADs, the transducers of tumor growth factor-β (TGFβ) signaling. TGFβs are known to play a role in cardiac development and in several aspects of cardiovascular physiology ranging from the effect on cardiomyocyte and vascular smooth muscle, and renal control of blood pressure^31^.

In summary, we discovered two AS variants on chromosomes 1p21 and 2q22. Associations of these and two previously reported AS variants with BAV, other congenital heart defects, aortic root size, and CAD, involve both cardiac developmental abnormalities and atherogenesis-like processes in the pathogenesis of AS. Further, we demonstrate that four CAD variants and one aortic root size variant associate with AS. While our genetic causal analysis does not support a generalized sharing of genetic risk between CAD and AS, it indicates that the shared risk factors of Lp(a) and non-HDL cholesterol contribute substantially to the frequent co-occurence of these diseases.

## Methods

### deCODE discovery study subjects

The Icelandic AS sample set included all patients diagnosed in the years 1983–2016 with AS at Landspitali – The National University Hospital (LUH) in Reykjavik, the only tertiary referral center in Iceland. Case status was assigned based on ICD-10 codes I35.0 or I35.2 for discharge diagnoses, or the relevant NOMESCO classification of surgical procedure codes FMA, FMSA, FMD or FMSD, and subcodes). A total of 2,609 cases were identified and of those 2,457 had available genotypes and were included in the analysis. The controls included 349,342 population controls from the Icelandic genealogical database and individuals recruited through different genetic studies at deCODE genetics. The study was approved by the Icelandic Data Protection Authority and the National Bioethics Committee of Iceland (approval no. VSNb2015030022/03.01 with amendments). All participating subjects donating biological samples signed informed consents. Personal identities of the participants and biological samples were encrypted by a third-party system approved and monitored by the Icelandic Data Protection Authority.

The deCODE genetics phenotype database contains extensive medical information on various diseases and traits. Cardiovascular phenotypes used for the purpose of the study included CAD (*N* = 37,782)^32^, heart failure (*N* = 10,480), ischemic stroke (*N* = 8,948)^33^, atrial fibrillation (*N* = 13,471)^34^, sick sinus syndrome (*N* = 3,310)^10^, high-degree atrioventricular block (*N* = 1,303), BAV (*N* = 208), atrial septal defect (*N* = 708), ventricular septal defect (*N* = 902), coarctation of aorta (*N* = 119), thoracic aortic aneurysm (TAA) and dissection (*N* = 500), hypertension (*N* = 54,974), type 2 diabetes (*N* = 11,448)^35^, and aortic root diameter (*N* = 19,506). The heart failure, high-degree atrioventricular block, coarctation of aorta, TAA and dissection, atrial septal defect, and ventricular septal defect sample sets were based on discharge diagnoses from LUH. Hypertension diagnoses were obtained from the Primary Health Care Clinics of the Reykjavik area, or from LUH. The BAV sample set included individuals with a documentation of BAV in an echocardiographic report from LUH between 1994 and 2015. Measurements of aortic root diameter were obtained from a database of 53,122 echocardiograms from 27,460 individuals performed at LUH between 1994 and 2015. Non-HDL cholesterol measurements (*N* = 136,326) were obtained from three of the largest clinical laboratories in Iceland: (i) LUH (hospitalized and ambulatory patients); (ii) the Laboratory in Mjódd, Reykjavík (ambulatory patients); and (iii) Akureyri Hospital, Regional Hospital in North Iceland, Akureyri (hospitalized and ambulatory patients)^32^. Blood pressure measurements (*N* = 125,647) were obtained from the Primary Health Care Clinics of the Reykjavik area. Measurements were adjusted for sex, year of birth, and age at measurement, and were subsequently standardized to have a normal distribution.

### Whole-genome sequencing and imputation

This study is based on whole-genome sequence data from 15,220 Icelanders participating in various disease projects at deCODE genetics. In addition, 151,677 Icelanders have been genotyped using Illumina SNP chips and genotype probabilities for untyped relatives are calculated based on Icelandic genealogy. The sequencing was done using Illumina standard TruSeq methodology to a mean depth of 35× (SD 8)^13^. Autosomal single-nucleotide polymorphisms (SNPs) and INDEL’s were identified using the Genome Analysis Toolkit version 3.4.0^36^. Information about haplotype sharing was used to improve variant genotyping, taking advantage of the fact that all sequenced individuals had also been chip-typed and long-range-phased^37^.

### Genotype imputation information

The informativeness of genotype imputation (imputation information) was estimated by the ratio of the variance of imputed expected allele counts and the variance of the actual allele counts:

$$\frac{{{\rm Var}(E(\theta |{\rm chip}\,{\rm data}))}}{{{\rm Var}(\theta )}},$$

where *θ* is the allele count. Here, Var(*E*(*θ*|chip data)) is estimated by the observed variance in the imputed expected counts and Var(*θ*) was estimated by *p*(1−*p*), where *p* is the allele frequency.

### Gene and variant annotation

Variants were annotated using Ensembl release 80 and Variant Effect Predictor version 2.8^38^. A total of 32.5 million variants passed the quality threshold and were imputed into 151,677 Icelanders who had been genotyped using Illumina chips.

### Adjusting for relatedness

To account for inflation in test statistics due to cryptic relatedness and stratification, we applied the method of LD score regression^39^. With a set of 1.1 M variants, we regressed the *χ*^2^ statistics from our GWA scan against LD score and used the intercept as a correction factor. The LD scores were downloaded from an LD score database (ftp://atguftp.mgh.harvard.edu/brendan/1k_eur_r2_hm3snps_se_weights.RDS; accessed 23 June 2015).

### Thresholds for genome-wide significance

The threshold for genome-wide significance was corrected for multiple testing with a weighted Bonferroni adjustment using as weights the enrichment of variant classes with predicted functional impact among association signals^14^. With 32,463,443 sequence variants being tested, the weights given in Sveinbjornsson et al.^14^ were rescaled to control the family-wise error rate. This yielded significance thresholds of 2.6 × 10^−7^ for high-impact variants (*N* = 8,464), 5.1 × 10^−8^ for moderate-impact variants (*N* = 149,983), 4.6 × 10^−9^ for low-impact variants (*N* = 2,283,889), 2.3 × 10^−9^ for other variants in Dnase I hypersensitivity sites (*N* = 3,913,058) and 7.9 × 10^−10^ for other variants (*N* = 26,108,039).

### Association analysis

Logistic or linear regression were used to test for the association of SNPs with binary or quantitative traits, respectively, treating disease status or quantitative trait as the response and allele counts from direct genotyping or expected genotype counts from imputation as covariates. To account for inflation in test statistics due to cryptic relatedness and stratification, we applied the method of LD score regression^39^. The estimated correction factor for AS based on LD score regression was 1.19 for the additive model.

### Genetic risk score

Weighted GRS for CAD (CAD-GRS*-all*) was generated based on previously reported CAD effect estimates (logOR) (see Supplementary Data 4). Variants used for the CAD-GRS*-lip* included a subset of variants in CAD-GRS that associated with non-HDL cholesterol (*P* < 1 × 10^−8^) in the Icelandic dataset, or are located at the *LPA* locus. The two *LPA* locus variants included in CAD-GRS*-lip* associate with lipoprotein(a) at *P* < 8 × 10^−90^ in Iceland.

### Genetic association replication studies

The Malmo Diet and Cancer Study (MDCS) is a community-based prospective cohort of middle-aged individuals from Southern Sweden. In total, 30,447 subjects attended a baseline exam in 1991–1996 when they filled out a questionnaire, underwent anthropometric measurements, and donated peripheral venous blood samples^40^. Prevalent or incident cases of AS were ascertained from nationwide hospital registers with high validity as described previously^19^. Genome-wide genotyping of single-nucleotide variants was performed using the Illumina Human Omni Express Exome BeadChip kit. Genotyping was performed in a nested case-cohort design including 15,362 subjects with complete data, of which 470 cases with incident AS. The SNP rs10455872 was genotyped in the entire cohort, with genotypes available in 28,722 subjects, including 613 cases with incident AS. Association with incident AS was tested in a case–control analysis utilizing logistic regression under an additive inheritance model adjusted for age and sex. Case–control matching was performed in SAS v9.4 with the greedy algorithm, matching 1 AS case to 1 population-based controls for sex, baseline age (<3 years age difference), year of baseline visit (within 3 years from visit), and requiring at least equal follow-up in controls. All participants were of European ancestry, confirmed by multidimensional scaling of genome-wide data. Informed consent was obtained from all participants and the study was approved by the Ethics Committee of Lund University, Sweden.

Patients from the greater Stockholm area with AS, BAV, or TAA were recruited as a part of the ASAP (the Advanced Study of Aortic Pathology) and Artist studies. The ASAP cohort consists of 429 patients undergoing aortic valve surgery at the Karolinska University Hospital (Stockholm, Sweden)^29,41^. The samples were genotyped on Illumina 610wQuad beadchips and approximately 588,400 SNPs were provided after quality control (QC). The Artist cohort consists of 406 samples genotyped with Omni-2.5 Quad beadchips on 2,443,180 SNPs. Imputation was performed using Impute2 from 1000G phase1 v3. Samples from the POLCA/Olivia cohorts were used as controls (a total of 1,295 individuals). POLCA consists of healthy 50-year-old men, free from coronary heart disease, recruited at random using the population registry. The POLCA samples were genotyped on Illumina 610kwQuad. The Olivia comprises both men and women with an age distribution 33–80 years. In Olivia, 670 control samples were genotyped on illumina 1M genotyping arrays. The vast majority of included control samples are of Scandinavian ancestry. Association analysis was performed using SNPTEST, with age, sex, and first 10 principal components as covariates. The study was approved by the Human Research Ethics Committee at Karolinska Institutet (ASAP study, ethical approval number 2006/784-31/1; Artist cohort, 2008/1771-31; POLCA/Olivia control samples, 03-491), Stockholm, Sweden.

The Norwegian Nord-Trøndelag Health Study (HUNT) is a population-based health survey conducted in the county of Nord-Trøndelag, Norway. Individuals were included at three different time points during approximately 20 years (HUNT1 (1984–1986), HUNT2 (1995–1997), and HUNT3 (2006–2008))^42^. At each time point, the entire adult population (≥20 years) was invited to participate by completing questionnaires, attending clinical examinations, and interviews. Taken together, the health studies include information from over 120,000 different individuals from Nord-Trøndelag. Biological samples including DNA have been collected for approximately 70,000 participants. AS was defined based on ICD-10 codes collected from local hospitals and out-patient clinics between 1999 and 2016. Cases were defined as individual with one or more ICD-10 codes specific for AS (“I35.0” or “I35.2”), whereas controls were all individuals without a code specific for AS. In total, 1,546 cases with AS and 24,235 controls genotyped with Illumina HumanCoreExome arrays were analyzed. Association analysis were conducted using EPACTS-3.3. The SNP-phenotype associations were modeled using the Firth Bias-Corrected Logistic Likelihood Ratio Test^43^, assuming an additive genetic model for genotyped markers and imputed genotypes. Models were adjusted for sex, birth year, genotyping batch, and four principal components (PCs). PCs were computed using PLINK. Individuals of non-European ancestry, based on principal components analysis (PCA) were excluded from the study. Additional filters applied to the analysis included minor allele count ≥10 and imputation *r*^2^ ≥ 0.3. Participation in the HUNT Study is based on informed consent, and the study has been approved by the Data Inspectorate and the Regional Ethics Committee for Medical Research in Norway.

In the years 2006–2010, the UK Biobank study recruited 502,647 individuals aged 37–76 years from across the country. All participants provided information regarding their health and lifestyle via touch screen questionnaires, consented to physical measurements, and agreed to have their health followed. They also provided blood, urine, and saliva samples for future analysis. UK Biobank has ethical approval from the Northwest Multi-Center Research Ethics Committee, and informed consent was obtained from all participants. Genotype imputation data was available for 487,409 individuals (May 2017 release), of which 408,658 were used in the analysis. The 408,658 individuals were selected as self-reported white British with similar genetic ancestry based on principal component analysis and with consistent reported and genetically determined gender. AS was defined according to ICD-10 codes I35.0 and I35.2, based on diagnoses codes a participant has had recorded across all their episodes in hospital, and CAD was defined as the codes I20.0, I21, I22, I25.0, I25.1, I25.2, and I25.9. The case–controls analysis was done using SNPTEST v2.5.2^44^ and the association with the GRS was tested using R v3.4.1^45^. In both cases, the analysis was adjusted for age, gender, and 20 principle components. To adjust for relatedness and remaining population stratification the *P* values were adjusted using genomic control adjustment, with adjustment factors *λ*_g_ estimated based on association analysis of 155,000 unrelated common variants. The estimated *λ*_g_'s were 1.023, 1.088, 1.029, and 1.013 for the analysis of AS, CAD, AS restricted to CAD and AS excluding CAD, respectively.

In the University of Michigan and Cardiovascular Health Improvement Study, we collected DNA from consented individuals with BAV from the Frankel Cardiovascular Center at the University of Michigan as part of the University of Michigan BAV registry or the Cardiovascular Health Improvement Project (CHIP). Patients were typically seen in clinic for aortic valve replacement or aortic aneurysm. DNA was isolated from peripheral blood lymphocytes. Four hundred and seventy-three BAV cases, 251 AS cases, and 809 TAA cases were successfully collected and genotyped. We identified potential controls from a surgical-based biobank, the Michigan Genomics Initiative (MGI), that were genotyped with the same GWAS array as cases. After excluding those with aortic disease, we performed age matching by requiring controls to have a birth year within −5 and +10 years of the case. From the available controls in the appropriate age and sex category, we selected the best ethnic match for each case and repeated the greedy algorithm until a control was selected for each case. We repeated the entire process so that 10 controls were selected for each AS and BAV case and 5 controls for each TAA case. All MGI research subjects provided informed consent. We performed genotyping using a GWAS+exome chip array (Illumina HumanCoreExome). To avoid any potential batch effects, cases and controls were genotyped using the same array in the same genotyping center (Sequencing and Genotyping core at the University of Michigan). Genotype calling was performed using GenTrain version 2.0 in GenomeStudio V2011.1 (Illumina) using identical cluster files for cases and controls. Samples with <98% genotype calls, evidence of gender discrepancy, and duplicates as well as individuals with non-European ancestry identified by plotting the first 10 genotype-driven principal components were excluded from further analysis. We performed variant-level QC by excluding variants that met any of the following criteria; variants with a cluster separation score <0.3, <98% genotype call rate, or deviation from Hardy–Weinberg equilibrium (*P* < 1 × 10^−5^). We phased the autosomal genotype data using SHAPEIT2^46^ and imputed variants from the Haplotype Reference Panel v1^47^ using minimac3^48,49^. We excluded poorly imputed variants with imputation R2 < 0.3. We performed single-variant association testing for BAV, TAA, and AS status using the Wald test based on logistic regression with age, sex, and the first four principal components as covariates using the EPACTS software (URL: http://csg.sph.umich.edu//kang/epacts/) for imputed dosages. All repository projects utilized for this study are approved by the University of Michigan, Medical School, Institutional Review Board, and informed consent was obtained from study participants.

In the BAVCon consortium, 452 sporadic self-reported Caucasian BAV cases were genotyped using the Illumina Omni-2.5 platform. One thousand eight hundred and thirty-four self-reported Caucasian population controls from the Framingham Heart Study genotyped using the Omni5 platform. Caucasian ancestry was further identified using PCA to detect clusters, filter outliers, and filter-related individuals both before and after merging cases and controls for association analysis. Principal components were included as covariates in association analysis. Further QC of the genotype data from both cohorts was performed using GenomeStudio and PLINK. After QC, we imputed additional genotypes against the 1,000 genomes reference (Phase 3) using IMPUTE2 to yield 7,913,553 genetic markers for an additive logistic regression model, adjusted for gender and age. This study has been approved by Partner’s in HealthCare Human Research Committee, and informed consent was obtained from study participants.

In the University of Texas Health Science Center, 765 patients of European descent with TAA or aortic dissections, were enrolled and genotyped^50^. A subset of these patients had BAV, *N* = 147. The study included 864 controls from US National Institute of Neurological Disorders and Stroke (NINDS)^51^. We imputed additional genotypes against 1,000 genomes reference (Phase 3), and for association analysis, we used additive logistic regression model accounting for gender and principal components^51^. Subjects of non-European ancestry according to multidimensional scaling were removed from the analysis. This study has been approved by Committee for the Protection of Human Subjects at UT Health Science Center at Houston, and informed consent was obtained from study participants.

### Identification of candidate causal variants and genes

Epigenome data from the NIH Roadmap Epigenome Mapping Consortium (http://egg2.wustl.edu/roadmap/data/byFileType/chromhmmSegmentations/ChmmModels/imputed12marks/jointModel/final/catMat/hg19_chromHMM_imputed25.gz) for 11 histone marks analyzed by chromatin immunoprecipitation with sequencing, together with open chromatin regions analyzed by DNase-seq, integrated into 25 discrete chromatin states through ChromHMM were downloaded^26^. Lead variants and those found in LD (*R*^2^ > 0.5) within the AS loci were then annotated for chromatin states involving regulatory functions, that is, EnhA (Active enhancer elements), EnhW (Weak enhancer elements), EnhTx (Enhancer marks coupled with transcription-associated histone marks), DNAse (Open chromatin configuration), TssA (Active transcription start site), PromUp/D (Regions upstream or downstream of promoter), PromP (Poised promoter), PromBiv (Bivalent promoters), Het (Heterochromatin), ReprPC (Polycomb-group repressed) while omitting states indicative of transcription status only (Tx3′, Tx5′, Tx, TxWk) or states characteristic of zinc-finger protein genes (ZNF/Rpts).

High-throughput 3C analysis (Hi-C) data for right ventricular and left ventricular tissue and aorta were obtained from an online repository: Functional Mapping and Annotation of Genome-Wide Association Studies (FUMA GWAS: http://fuma.ctglab.nl/)^52^. It uses public datasets compiled by Schmitt et al.^28^ in order to identify structural interactions of enhancers with genes (Hi-C compendium: https://www.ncbi.nlm.nih.gov/geo/query/acc.cgi?acc = GSE87112)

Gorpipe^53^ was employed to query the data and then imported into R for further analysis and plotting making use of base functions (R 3.4.1). The Hi-C data are binned into intervals of 40 kb which are indicated as gray horizontal lines in lower panels of Fig. 1a, b, and their midpoints were then used to draw arcs from one midpoint to another for interactions at false-discovery rate <1 × 10^−10^. Intersection between chromatin interaction intervals and promoters was based on Refseq annotated transcription start sites.

### Data availability

The Icelandic population WGS data has been deposited at the European Variant Archive under accession code PRJEB8636. The authors declare that the data supporting the findings of this study are available within the article, its Supplementary Data files and upon request.

## Electronic supplementary material

Supplementary Information

Peer Review File

Description of Additional Supplementary Files

Supplementary Data 1

Supplementary Data 2

Supplementary Data 3

Supplementary Data 4

Supplementary Data 5

Supplementary Data 6

Supplementary Data 7

## References

[1] Danielsen R, Aspelund T, Harris TB, Gudnason V (2014). The prevalence of aortic stenosis in the elderly in Iceland and predictions for the coming decades: the AGES–Reykjavík study. Int. J. Cardiol..

[2] Osnabrugge RLJ (2013). Aortic stenosis in the elderly: disease prevalence and number of candidates for transcatheter aortic valve replacement: a meta-analysis and modeling study. J. Am. Coll. Cardiol..

[3] Vahanian A, Joint Task Force on the Management of Valvular Heart Disease of the European Society of Cardiology (ESC (2012). Guidelines on the management of valvular heart disease (version 2012). Eur. Heart J..

[4] Dweck MR, Boon NA, Newby DE (2012). Calcific aortic stenosis. J. Am. Coll. Cardiol..

[5] Siu SC, Silversides CK (2010). Bicuspid aortic valve disease. J. Am. Coll. Cardiol..

[6] Thanassoulis G (2013). Genetic associations with valvular calcification and aortic stenosis. N. Engl. J. Med..

[7] Arsenault BJ (2014). Lipoprotein(a) levels, genotype, and incident aortic valve stenosis: a prospective mendelian randomization study and replication in a case–control cohort. Circ. Cardiovasc. Genet..

[8] Clarke R (2009). Genetic variants associated with Lp(a) lipoprotein level and coronary disease. N. Engl. J. Med..

[9] Helgadottir A (2012). Apolipoprotein(a) genetic sequence variants associated with systemic atherosclerosis and coronary atherosclerotic burden but not with venous thromboembolism. J. Am. Coll. Cardiol..

[10] Holm H (2011). A rare variant in MYH6 is associated with high risk of sick sinus syndrome. Nat. Genet..

[11] Thorolfsdottir RB (2017). A missense variant in PLEC increases risk of atrial fibrillation. J. Am. Coll. Cardiol..

[12] Bjornsson, T. et al. A rare missense mutation in MYH6 confers high risk of coarctation of the aorta. Preprint at http://www.biorxiv.org/content/early/2017/08/29/180794 (2017).

[13] Gudbjartsson DF (2015). Large-scale whole-genome sequencing of the Icelandic population. Nat. Genet..

[14] Sveinbjornsson G (2016). Weighting sequence variants based on their annotation increases power of whole-genome association studies. Nat. Genet..

[15] Wild PS (2017). Large-scale genome-wide analysis identifies genetic variants associated with cardiac structure and function. J. Clin. Invest..

[16] Guauque-Olarte S (2015). Calcium signaling pathway genes *RUNX2* and *CACNA1C* are associated with calcific aortic valve disease. Circ. Cardiovasc. Genet..

[17] Deloukas P, CARDIoGRAMplusC4D Consortium (2013). Large-scale association analysis identifies new risk loci for coronary artery disease. Nat. Genet..

[18] Carabello BA (2013). Introduction to aortic stenosis. Circ. Res..

[19] Smith JG (2014). Association of low-density lipoprotein cholesterol-related genetic variants with aortic valve calcium and incident aortic stenosis. JAMA.

[20] Andell P (2017). Epidemiology of valvular heart disease in a Swedish nationwide hospital-based register study. Heart.

[21] Nikpay M (2015). A comprehensive 1,000 Genomes-based genome-wide association meta-analysis of coronary artery disease. Nat. Genet..

[22] Howson JMM (2017). Fifteen new risk loci for coronary artery disease highlight arterial-wall-specific mechanisms. Nat. Genet..

[23] Wilton E, Jahangiri M (2006). Post-stenotic aortic dilatation. J. Cardiothorac. Surg..

[24] Gertow K (2012). Identification of the BCAR1-CFDP1-TMEM170A locus as a determinant of carotid intima–media thickness and coronary artery disease risk. Circ. Cardiovasc. Genet..

[25] Mele M (2015). The human transcriptome across tissues and individuals. Science.

[26] Kundaje A, Roadmap Epigenomics Consortium (2015). Integrative analysis of 111 reference human epigenomes. Nature.

[27] Ernst J, Kellis M (2012). ChromHMM: automating chromatin-state discovery and characterization. Nat. Methods.

[28] Schmitt AD (2016). A compendium of chromatin contact maps reveals spatially active regions in the human genome. Cell Rep..

[29] Jackson V (2011). Bicuspid aortic valve leaflet morphology in relation to aortic root morphology: a study of 300 patients undergoing open-heart surgery. Eur. J. Cardiothorac. Surg..

[30] Rossebø AB (2008). Intensive lipid lowering with simvastatin and ezetimibe in aortic stenosis. N. Engl. J. Med..

[31] Azhar M (2003). Transforming growth factor beta in cardiovascular development and function. Cytokine Growth Factor. Rev..

[32] Helgadottir A (2016). Variants with large effects on blood lipids and the role of cholesterol and triglycerides in coronary disease. Nat. Genet..

[33] Gretarsdottir S (2008). Risk variants for atrial fibrillation on chromosome 4q25 associate with ischemic stroke. Ann. Neurol..

[34] Gudbjartsson DF (2007). Variants conferring risk of atrial fibrillation on chromosome 4q25. Nature.

[35] Steinthorsdottir V (2014). Identification of low-frequency and rare sequence variants associated with elevated or reduced risk of type 2 diabetes. Nat. Genet..

[36] McKenna A (2010). The Genome Analysis Toolkit: a MapReduce framework for analyzing next-generation DNA sequencing data. Genome Res..

[37] Kong A (2008). Detection of sharing by descent, long-range phasing and haplotype imputation. Nat. Genet..

[38] McLaren W (2010). Deriving the consequences of genomic variants with the Ensembl API and SNP Effect Predictor. Bioinformatics.

[39] Bulik-Sullivan BK (2015). LD score regression distinguishes confounding from polygenicity in genome-wide association studies. Nat. Genet..

[40] Smith JG, Platonov PG, Hedblad B, Engström G, Melander O (2010). Atrial fibrillation in the Malmö Diet and Cancer study: a study of occurrence, risk factors and diagnostic validity. Eur. J. Epidemiol..

[41] Folkersen L (2010). Association of genetic risk variants with expression of proximal genes identifies novel susceptibility genes for cardiovascular disease. Circ. Cardiovasc. Genet..

[42] Krokstad S (2013). Cohort Profile: the HUNT Study, Norway. Int. J. Epidemiol..

[43] Ma C, Blackwell T, Boehnke M, Scott LJ, GoT2D investigators. (2013). Recommended joint and meta-analysis strategies for case–control association testing of single low-count variants. Genet. Epidemiol..

[44] Marchini J, Howie B, Myers S, McVean G, Donnelly P (2007). A new multipoint method for genome-wide association studies by imputation of genotypes. Nat. Genet..

[45] R Development Core Team. (2008). R: a language and environment for statistical computing. R. Found. Stat. Comput..

[46] Delaneau O, Marchini J, Zagury JF (2011). A linear complexity phasing method for thousands of genomes. Nat. Methods.

[47] McCarthy S (2016). A reference panel of 64,976 haplotypes for genotype imputation. Nat. Genet..

[48] Howie B, Fuchsberger C, Stephens M, Marchini J, Abecasis GR (2012). Fast and accurate genotype imputation in genome-wide association studies through pre-phasing. Nat. Genet..

[49] Fuchsberger C, Abecasis GR, Hinds D (2015). A. minimac2: faster genotype imputation. Bioinformatics.

[50] Prakash SK (2010). Rare copy number variants disrupt genes regulating vascular smooth muscle cell adhesion and contractility in sporadic thoracic aortic aneurysms and dissections. Am. J. Hum. Genet..

[51] LeMaire SA (2011). Genome-wide association study identifies a susceptibility locus for thoracic aortic aneurysms and aortic dissections spanning FBN1 at 15q21.1. Nat. Genet..

[52] Watanabe K, Taskesen E, van Bochoven A, Posthuma D (2017). Functional mapping and annotation of genetic associations with FUMA. Nat. Commun..

[53] Guðbjartsson H (2016). GORpipe: a query tool for working with sequence data based on a Genomic Ordered Relational (GOR) architecture. Bioinformatics.

