# Supplementary material for: Genome-wide analysis yields new loci associating with aortic valve stenosis
Source: Nat Commun. 2018 Mar 7;9:987. doi: 10.1038/s41467-018-03252-6 (PMC5840367; doi:10.1038/s41467-018-03252-6)
Supplement: Supplementary file 1 — Supplementary Information [file 41467_2018_3252_MOESM1_ESM.pdf]

Supplementary Information

Genome-wide analysis yields new loci associating with aortic valve stenosis

Helgadottir, *et.al.*

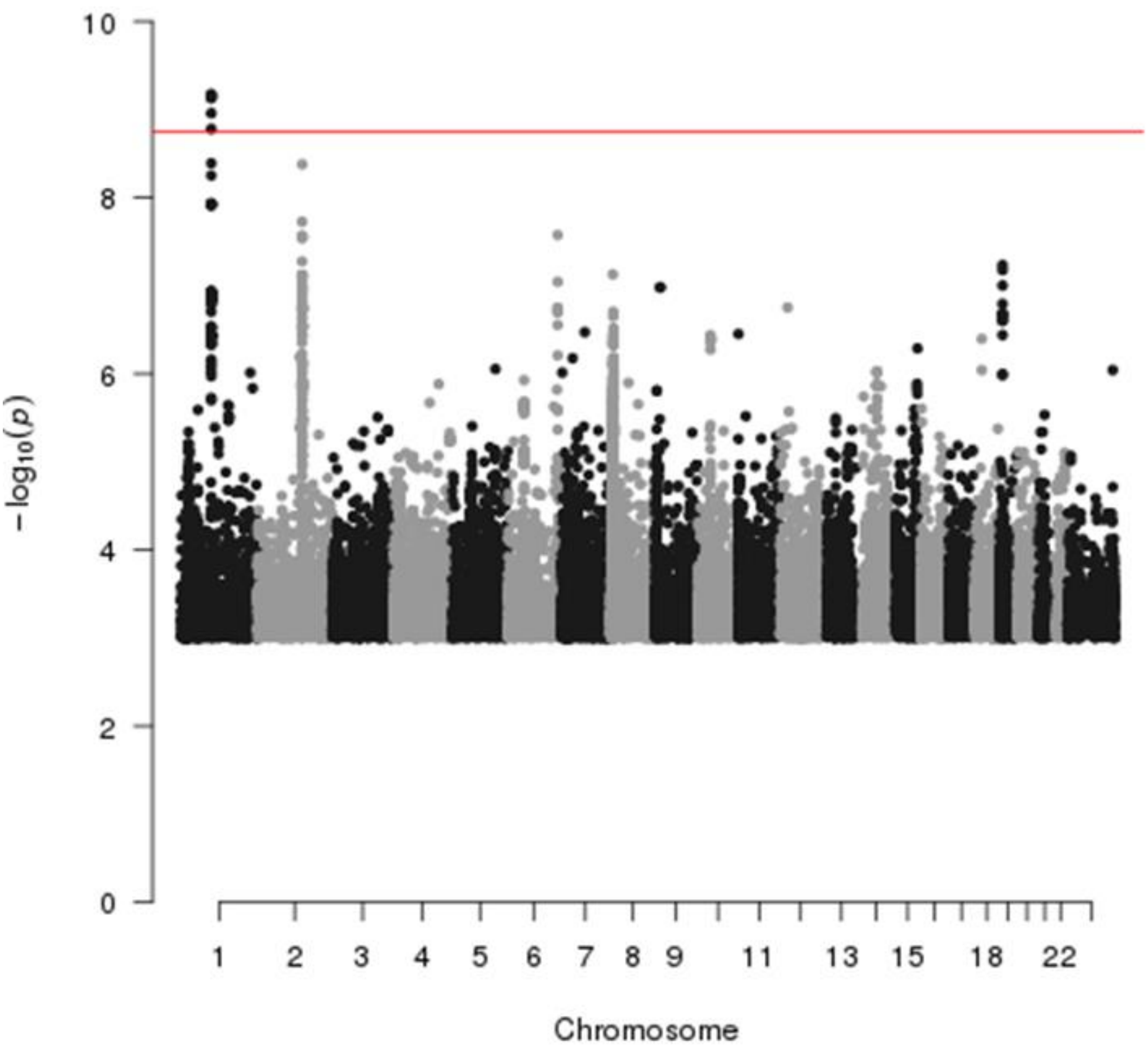

Supplementary Fig. 1

Manhattan plot for the discovery genome-wide association analysis. The red horizontal line indicates the genome-wide significance threshold for low-impact variants ($P < 4.6 \times 10^{-9}$).

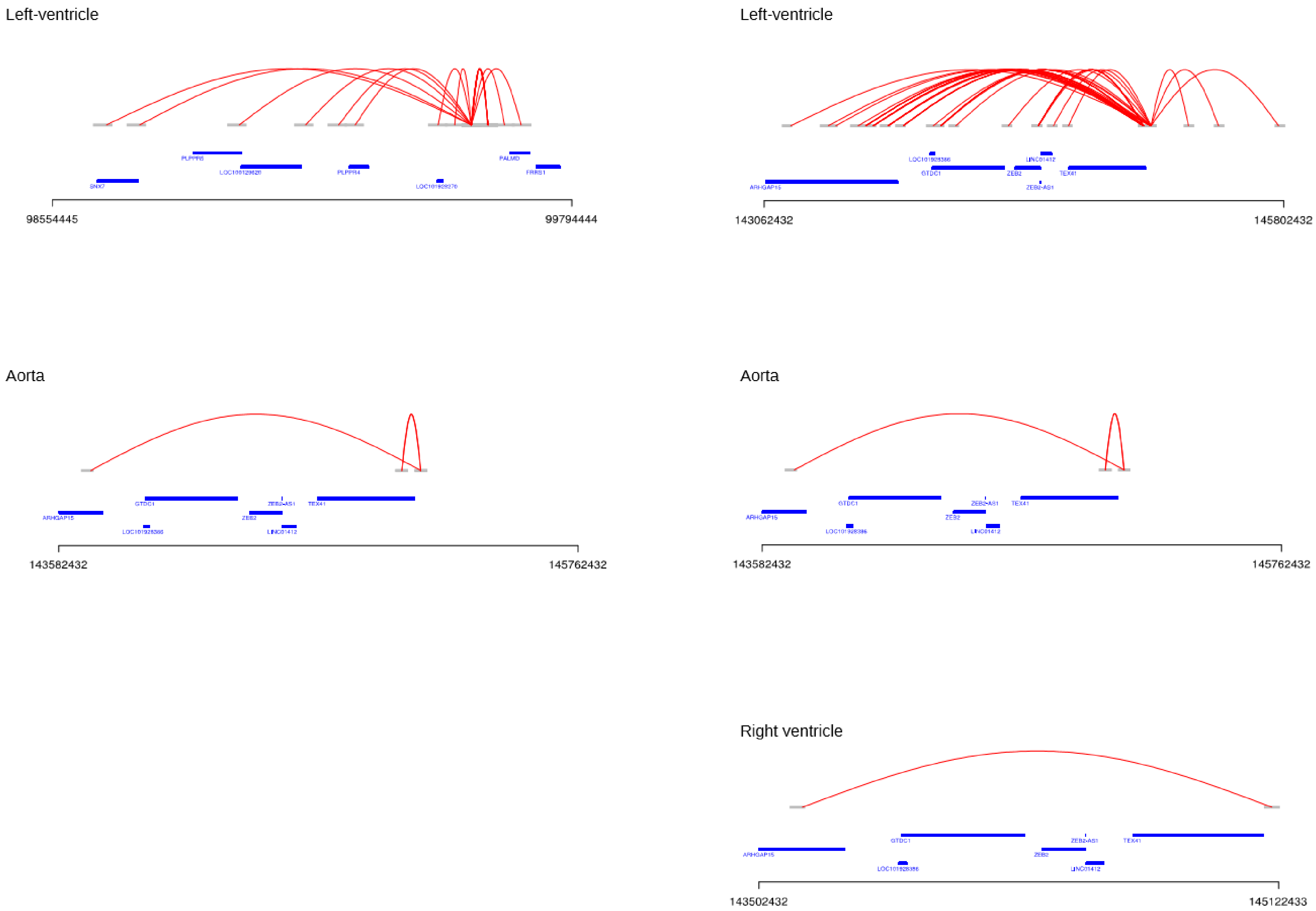
Left-ventricle
98554445
99794444
Left-ventricle
143062432
145802432
Aorta
143582432
145762432
Aorta
143582432
145762432
Right ventricle
143502432
145122433

Supplementary Fig. 2

Shown are all chromatin interactions observed for the regulatory regions, to which AS risk variants at the *PALMD* locus (on the left-hand side) and at the *TEX41* locus (on the right-hand side) map to.

The chromatin interactions are shown for left- and right ventricular heart, and aorta tissue samples.

At the *PALMD* locus no chromatin interactions were observed in right ventricular tissue.
