# Supplementary material for: Genome-wide analysis yields new loci associating with aortic valve stenosis
Source: Nat Commun. 2018 Mar 7;9:987. doi: 10.1038/s41467-018-03252-6 (PMC5840367; doi:10.1038/s41467-018-03252-6)
Supplement: Supplementary file 3 — Description of Additional Supplementary Files [file 41467_2018_3252_MOESM3_ESM.pdf]

Description of Supplementary Files

File Name: Supplementary Data 1
Description: Replication of variants with suggestive association with aortic stenosis in Iceland.

File Name: Supplementary Data 2
Description: Association of aortic stenosis variants with other cardiovascular phenotypes.

File Name: Supplementary Data 3
Description: Association of aortic stenosis variants with traditional cardiovascular risk factors.

File Name: Supplementary Data 4
Description: The effect of established coronary artery disease (CAD) variants on CAD and aortic valve stenosis (AS).

File Name: Supplementary Data 5
Description: The effect of established coronary artery disease (CAD) variants on aortic valve stenosis (AS) in Iceland and UK-Biobank.

File Name: Supplementary Data 6
Description: The association of CAD genetic risk score with aortic valve stenosis.

File Name: Supplementary Data 7
Description: Association of reported aortic root size variants on aortic valve stenosis.
